# Health care Experiences of Educated Young Adults With Blindness in the Digital Age: Qualitative Study

**DOI:** 10.2196/79587

**Published:** 2025-11-21

**Authors:** Junling Zhao, Can Su, Xiji Zhu, Cong Cai, Wei Liu, Xiaochen Ma

**Affiliations:** 1 China Center for Health Development Studies, Peking University, 112 Shu Wahh Building, 38 XueYuan Road, Haidian District, Beijing, 100191, China, 86 10-8280-57; 2 School of Public Health, Peking University, Beijing, China; 3 National Health Commission Key Laboratory of Health System Reform and Governance, Peking University, Beijing, China; 4 Business School, Hitotsubashi University, Kunitachi, Japan; 5 Journalist and Communication School, University of China Academy of Social Science, Beijing, China; 6 Business School, Central University of Finance and Economics, Beijing, China

**Keywords:** digital health technologies, young adults, blindness, health care accessibility, health equity, empowerment, exclusion, digital divide, qualitative study, China

## Abstract

**Background:**

The rapid advancement of digital health technologies (DHTs) offers substantial potential for improving health care access; however, it simultaneously risks exacerbating existing inequities for marginalized populations. Previous research on the digital divide has often treated individuals with blindness as a homogenous group, primarily focusing on barriers related to digital access and skills. However, less is known about the nuanced experiences of specific subgroups, such as educated and digitally literate young adults. This study focuses on this demographic to understand how their advanced digital capabilities interact with systemic and infrastructural barriers in health care.

**Objective:**

This study aimed to explore the lived health care experiences of educated young adults with blindness in China, specifically identifying how DHTs simultaneously contribute to their empowerment and exclusion.

**Methods:**

Eligible participants were educated young adults with blindness in China (aged 18‐30 years, Mandarin speakers, smartphone users, and holding or pursuing higher education). A total of 12 semistructured interviews were conducted in Mandarin during September 2024. All interviews were audio-recorded and transcribed verbatim. An inductive thematic analysis was used to interpret the data and identify key themes.

**Results:**

Participants’ experiences highlighted an “empowered but excluded” dynamic. Seven key themes emerged, categorized into empowerment and exclusion. Empowerment themes included: (1) digital platforms empowering self-management and health care access, where DHTs enabled independent appointment booking and access to comprehensive health information; and (2) digital platforms empowering for finding medical visit companions, facilitating the discovery of companions for physical and emotional support. Exclusion themes comprised: (3) inaccessible online appointment systems, due to noninclusive designs; (4) inaccessible health care environments and information formats, stemming from nonaccessible self-service machines and written materials; (5) lack of provider competencies in respecting patient autonomy, as providers often assumed digital incompetence; (6) data privacy and security concerns, heightened by increased digitalization and reliance on assistive tools; and (7) challenges related to the quality and consistency of online companion support, highlighting the limitations of platform-based assistance.

**Conclusions:**

Our findings reveal an “empowered but excluded” dynamic: the potential for digital empowerment and enhanced independence is often curtailed by systematic barriers. Addressing this necessitates a multifaceted approach: enhancing technological accessibility through robust standards adherence and inclusive co-design processes; improving health care provider competencies in patient-centered care via targeted training; and empowering educated young adults with blindness by building their capacity for self-determination to achieve equitable health care access.

## Introduction

Digital health technologies (DHTs), encompassing a wide array of tools from mHealth apps and telemedicine to artificial intelligence, hold transformative potential for health care worldwide [1,2]. By expanding access to care, enhancing patient engagement, and improving the efficiency of diagnostic and treatment pathways, these technologies offer significant opportunities to build more accessible, affordable, and equitable health systems [3-6]. The World Health Organization defines digital health as “the field of knowledge and practice associated with the development and use of digital technologies to improve health.” [7] This broad concept includes not only established eHealth domains but also emerging areas such as big data analytics and the Internet of Things, reflecting its integral role in modern health care.

However, the benefits of digital health are not universally realized and are not distributed equally [8]. Factors such as digital literacy, access to devices and internet, socioeconomic status, cultural relevance, and community context influence who benefits from digital health solutions [9,10]. In fact, digital literacy and internet connectivity have been termed “super social determinants of health” because of their foundational influence on all other determinants of health in the digital age [11]. The rapid digitization of health care may widen health disparities if solutions are not developed with these determinants in mind [12]. Growing evidence suggests that the digital transformation in health care may exacerbate existing health inequities, creating new barriers for marginalized populations including persons with disabilities, patients of racial or ethnic minority groups, those with limited language proficiency, and people with low socioeconomic status [13-15].

Previous research on the digital divide and health care access for vulnerable groups has illuminated various forms of exclusion, such as the inaccessibility of health websites and mobile apps, often due to a lack of distinguishable button features, inaccessible content, or the absence of assistive technology integration [16,17]. For individuals with blindness specifically, existing literature frequently points to significant challenges in interacting with visually-oriented digital environments [18]. Crucially, much of this prior research tends to homogenize the experiences of vulnerable populations [19], overlooking the nuanced realities and varying adaptive capacities within specific subgroups. This oversight means that while broad challenges are identified, the potential for certain segments of vulnerable communities to navigate and even leverage digital tools remains underexplored.

Within this context, educated and digitally literate young adults with blindness represent a critically overlooked and underexplored subgroup. For the purpose of this study, we define our participant cohort as follows: “young adults” refers to individuals aged 18 to 30 years [20], a generation broadly considered digitally native; “educated” refers to individuals who have received or are currently pursuing higher education (including associate’s, Bachelor’s, Master’s, or doctoral degrees); and “blindness” is defined according to the World Health Organization criteria of a presenting visual acuity of less than 3/60 in the better eye [21]. This cohort embodies a central paradox: they are a digitally native generation, often exhibiting a greater willingness and capacity to adopt new technologies and engage in digital transformation through exploratory learning. The proliferation of smartphones equipped with assistive features like screen readers and voice assistants theoretically holds significant promise for enhancing their independence. However, their entire digital experience is mediated by these assistive technologies, rendering them uniquely vulnerable to design and usability flaws in mainstream applications. The existing literature, by not adequately differentiating within the community with blindness, fails to capture the unique dynamic of empowerment and exclusion experienced by this specific subgroup. This study addresses the critical gap by proposing that a segment of high-literacy individuals with blindness, through personal effort and adaptive strategies, can indeed mitigate some impacts of the digital divide, a nuanced perspective often underestimated in studies that generalize vulnerabilities. Understanding this internal heterogeneity is paramount for developing genuinely effective and equitable digital health solutions.

China offers a uniquely relevant context for exploring these complex issues. It boasts one of the world*’*s largest internet user bases, exceeding 1.1 billion individuals as of 2024 [22], with extensive access to a variety of internet-based services, including health care [23]. Concurrently, China is home to one of the largest populations with disabilities in the world, including nearly 10 million who are blind [24], a significant proportion of whom are young. While some studies in China have identified health care barriers for visually impaired individuals, such as difficulties with registration, navigation, and understanding treatment processes [18], the majority of empirical studies on digital health access have tended to focus on older adults or persons with disabilities in general. These studies offer valuable broad overviews but often do not provide in-depth insights into the specific experiences of educated young adults with blindness navigating both empowerment and exclusion in a rapidly digitizing health care system. The unique combination of a highly developed digital infrastructure and a large young population with blindness in China provides invaluable insights into how accessibility challenges persist and manifest even amid advanced technological environments, underscoring the urgency for inclusive design.

To address this gap, this qualitative study aims to comprehensively explore the lived experiences of educated and digitally literate young adults with blindness in China as they access health care services in the digital age. A qualitative methodology is uniquely suited to capture the rich, in-depth narratives of these interactions, uncovering the nuanced facilitators and barriers that quantitative methods might miss. This nuanced understanding of their lived experiences with the digital health ecosystem can inform policy developments and improve clinical practices in promoting digital health equity.

## Methods

### Study Design

We used a qualitative design to gain an in-depth understanding of how educated and digitally literate young adults with blindness navigate health care access with the assistance of DHTs. This approach was chosen to capture the rich, subjective lived experiences and perceptions of participants, offering deep insights into how they interpret their personal encounters, construct their realities, and attribute meaning to their experiences within a rapidly digitizing health care ecosystem [25]. A qualitative methodology is particularly appropriate for exploring complex social phenomena where individual perspectives are central to uncovering the underlying dynamics of empowerment and exclusion. This study adheres to the Consolidated Criteria for Reporting Qualitative Research (COREQ) guidelines (Checklist 1) [26].

### Participants and Recruitment

#### Participants

This study focused on educated young adults with blindness who actively use smartphones and digital platforms to access health care services. Participants were selected based on the following inclusion and exclusion criteria (Textbox 1).

Textbox 1. **Inclusion and exclusion criteria.**

**Inclusion criteria**

- Citizens and residents of China
- Mandarin speakers
- Young adults aged 16 to 36 years
- Higher education (associate’s degree, Bachelor’s degree, or higher)
- Capable of independently operating at least 1 digital device (eg, smartphone or computer)
- Individuals with blindness (presenting visual acuity worse than 3/60 in the better eye, based on World Health Organization standards [21])

**Exclusion criteria**

- No experience seeking health care services within the past 2 years
- Unwilling to participate or unable to clearly articulate their experiences
- Failure to meet any of the defined inclusion criteria

#### Recruitment and Sample Size

A purposive snowball sampling approach was used to recruit participants. Initially, participants were selected from online communities and social media platforms serving people with blindness in China. Initial recruitment was facilitated by author CC (who is also a highly educated adult with blindness), who posted the study invitation in several WeChat groups dedicated to information exchange and community building among the population with blindness. Interested and eligible individuals were then contacted directly by the author for screening. Following their interview, initial participants were asked to refer peers in their network who also met the study criteria, thus generating the subsequent snowball sample.

A total of 12 participants were recruited for this study. In qualitative research, the sample size was determined by the principle of data saturation, not statistical generalizability. This approach is supported by findings from Guest et al [27], which indicate that 12 interviews are often sufficient to reach thematic saturation in a relatively homogeneous sample. Our analysis showed a similar pattern: over 70% of themes were identified within the first 6 interviews, and the primary core themes were established by the 10th interview. To confirm saturation, 2 additional interviews were conducted, which yielded no new core themes. Therefore, the final sample of 12 participants was considered sufficient for a comprehensive analysis.

### Data Collection

Semistructured interviews were conducted in Mandarin Chinese during September 2024 by 1 author (JZ), a female PhD student trained in qualitative research methods. The interviewer had no prior relationship with the participants, which helped minimize biases and address potential ethical concerns. All interviews were carried out remotely using the Tencent Meeting (Tencent Technology Co Ltd) videoconferencing platform. Tencent Meeting was selected due to the necessity for remote data collection during the COVID-19 outbreak and its status as a mainstream, accessible, and free online conferencing tool widely used in mainland China [28,29]. This ensured both the safety of participants and researchers and provided a familiar and convenient platform for our digitally literate participants with blindness. Before the interviews, participants were provided with detailed information about the study’s purpose, procedures, and the expected time commitment.

A topic guide with open-ended questions (Multimedia Appendix 1) was used during interviews to ensure comprehensive coverage of relevant topics and allow participants to freely elaborate on their experiences. Follow-up questions were posed as needed to clarify responses and gather more detailed information on participants’ perspectives. The interview questions were developed by reviewing the existing literature and absorbing expert opinions. To ensure validity, the guide was pretested by 2 educated adults with blindness (who were not included in the final sample) and revised based on their feedback. Participants were initially asked to share their personal background and how they became blind. Subsequently, they were prompted to describe their past and present experiences in accessing health care services, while the third part focused on the perceived benefits and challenges of using digital tools, including how such technologies empowered or hindered their access to health care. Follow-up questions were tailored to participants’ responses to encourage deeper elaboration. Finally, participants were invited to share any additional thoughts or address overlooked aspects before concluding the interview. The interviewer took field notes during the interviews to supplement the data and highlight key moments [30]. A total of 12 interviews were conducted, with durations ranging from 35 to 90 minutes (mean 55.0, SD 18.5). All interviews were audio-recorded with participants’ permission, transcribed verbatim, and checked by participants.

### Ethical Considerations

This study received ethical approval from the Peking University Institutional Review Board (IRB00001052-22097). Due to the participants’ blindness, a verbal informed consent process was meticulously followed. Before each interview, participants were thoroughly informed about the study’s purpose, procedures, their right to withdraw at any time without penalty, the voluntary nature of their participation, and the measures taken to ensure confidentiality. Oral consent was obtained after ensuring that participants fully understood all aspects of the study, and this consent was audio-recorded as part of the interview. To protect the privacy and confidentiality of participants, strict measures were implemented. All data collected, including interview transcripts and audio recordings, were anonymized immediately upon transcription by removing direct identifiers such as names, specific locations, or any other potentially identifying information. Pseudonyms were assigned to participants to ensure their anonymity in all research outputs. All data were stored securely on password-protected university servers accessible only to the research team. Participants received compensation ranging from 60 to 100 RMB (US $8.40 to $14.00) for their time and participation. We confirm that no images or other materials that could identify individual participants are included in this paper or any supplementary materials. All procedures involving human subjects were conducted in accordance with the ethical standards of the institutional and national research committee and with the Helsinki Declaration.

### Data Analysis

This study used thematic analysis, a flexible and powerful method for systematically generating robust findings by “identifying, analyzing, and reporting patterns (themes) within data” [31]. Following the inductive qualitative thematic analysis approach outlined by Braun and Clarke [31,32], our data analysis encompassed 3 phases: reading, coding, and theming, informed by practical thematic analysis guidelines [33].

The reading phase commenced with the transcription of recorded interviews by 1 author (JZ), which were subsequently verified by the participants. The translated interview transcripts were then imported into the qualitative data analysis software MAXQDA 24 (VERBI GmbH) to facilitate the analytical process. During this phase, the researchers achieved extensive familiarization with the data through repeated readings.

The coding phase began with initial code development and involved a systematic and iterative process. One researcher (JZ) initiated the process by assigning descriptive codes line-by-line to segments of the interview transcripts using MAXQDA 24. These codes were generated inductively, emerging organically from a close reading of the text. They represented specific concepts, ideas, or experiences directly relevant to the study’s objectives, aiming to capture the richness of participants’ perspectives in their own words. To ensure academic rigor and reliability, a second researcher (CS) independently analyzed 30% of the uncoded interview transcripts, generating her own list of key themes without any influence from JZ. After this blind coding process, the codes were discussed and compared among all authors. Code definitions were refined, and a shared codebook was developed. This iterative process involved reviewing and revising codes, merging similar concepts, and resolving discrepancies, ultimately ensuring a comprehensive and aligned approach to the remaining data [34].

The theming phase involved synthesizing these refined codes into broader, overarching themes that addressed the research questions [30,35]. Throughout the entire data analysis process, particular attention was paid to the concept of data saturation. Discussions regarding saturation began during the initial reading phase and continued iteratively throughout coding and theming to ensure that no new information was emerging and that the themes were well-developed and grounded in the data.

## Results

### Participants' Characteristics

A total of 12 educated and digitally literate young participants with blindness were included in this qualitative study (Table 1). The average age was 25.4 (SD 2.2) years, more than half were female (7/12, 58%), and most experienced blindness from an early age (9/12, 75%; aged <6 y). Reflecting the inclusion criteria, all participants were currently pursuing or had completed higher education: 17% (2/12) held junior college degrees, while 83% (10/12) had completed or were pursuing Bachelor’s degrees or higher. In terms of occupation, 58% (7/12) were employed, with the remaining 42% (5/12) being students or unemployed. Half of the participants (6/12, 50%) resided in first-tier cities, with the remaining half evenly distributed between new first-tier or second-tier and third-tier or below cities (3/12, 25% each). The most common reasons for seeking health care were acute conditions and injury treatment (6/12, 50%), followed by chronic and skin conditions (4/12, 33%).

**Table 1.** Demographic information of educated young adults with blindness (N=12).

Characteristics	Value
Age (y), mean (SD)	25.4 (2.2)
Sex, n (%)	
Male	5 (42)
Female	7 (58)
Age of blindness onset (y), n (%)	
Congenital or early onset (0‐5)	9 (75)
Acquired (>6)	3 (25)
Education, n (%)	
Junior college	2 (17)
Bachelor’s degree or higher (completed or in-progress)	10 (83)
Occupation, n (%)	
Employed	7 (58)
Students or unemployed	5 (42)
Residence (city tier), n (%)	
First-tier	6 (50)
New first-tier or second-tier	3 (25)
Third-tier and below	3 (25)
Primary health care visits, n (%)	
Acute conditions and injury treatment	6 (50)
Chronic and skin conditions	4 (33)
General check-ups	1 (8)
Gynecological care	1 (8)

### Overarching Category: Experiences of Empowerment but Exclusion in Digital Health Care

Participants’ experiences navigating health care in the digital age were rich and multifaceted, consistently revealing a complex dynamic of both empowerment and exclusion. Our thematic analysis yielded 7 key themes, which are presented under 2 overarching categories: empowerment (reflecting how digital technologies enhance autonomy and access), and exclusion (highlighting persistent barriers and unmet potentials in digital health care; Table 2).

**Table 2.** Overview of themes.

Overarching category and theme	Summary of key points identified
Empowerment	
Digital platforms empowering self-management and health care access	DHTs^a^ enabled participants to independently book appointments, reducing wait times and enhancing efficiency. These platforms also provided diverse and comprehensive health information, fostering self-advocacy and proactive health management.
Digital platforms empowering for finding medical visit companions	DHTs facilitated the discovery of medical companions, improving access to services and fostering a sense of independent navigation. This assistance provided both physical navigation and emotional support during hospital visits.
Exclusion	
Inaccessible online appointment systems	Online appointment systems often lacked inclusive booking options and featured cluttered interfaces not optimized for screen readers, limiting access for individuals with blindness despite the general shift to digital platforms.
Inaccessible health care environments and information formats	The absence of accessible interfaces on self-service machines (eg, for check-in, payment, and prescription pickup) and the lack of accessible formats for written materials (eg, laboratory reports) created significant barriers within hospital environments.
Lack of provider competencies in respecting patient autonomy	Provider assumptions of digital incompetence led to communication being directed at sighted companions, undermining patient autonomy and reinforcing stereotypes, despite patients’ digital literacy.
Data privacy and security concerns	The increased digitalization of health services heightened concerns over data breaches, making privacy harder to maintain. Complex interfaces and the use of voice-based assistive tools in public settings further complicated privacy management.
Challenges related to the quality and consistency of online companion support	While enabling, reliance on online platforms for companions introduced specific challenges related to the inconsistent quality and limited capabilities of support, often lacking emotional connection and accountability.

a DHT: digital health technology.

### Empowerment: Digital Technologies Fostering Access

#### Digital Platforms Empowering Self-Management and Health Care Access

All 12 participants in this study demonstrated a high level of digital engagement, routinely using smartphones and screen reader technology to overcome accessibility challenges in daily life, extending their digital practices into areas such as information seeking, learning, and social interaction. The most frequently used applications include WeChat, Rednote (xiaohongshu in Chinese), TikTok (Douyin in Chinese), Bilibili, and Xianyu, which are popular platforms in China for social networking, content sharing, and e-commerce. This digital proficiency directly translated into enhanced health care engagement.

Participants reported using digital platforms to access health care services and information, including managing appointments and consulting health-related content online. In participants’ views, digital platforms offer two key advantages: (1) they provide a wealth of diverse and comprehensive information, surpassing traditional word-of-mouth referrals; and (2) they enable users to access this information with temporal and spatial flexibility, offering greater convenience compared to time- and location-bound methods. This enhanced access to information did more than improve convenience; it facilitated a fundamental shift from passive reliance on others to proactive self-advocacy. Participants perceived this newfound ability to independently seek out and act on information as a powerful form of self-expression and a significant gain in personal freedom. For instance, one participant described how digital platforms enabled her to proactively seek mental health support tailored to her needs:

I posted on Rednote saying that I am blind and looking for a psychiatrist who does not discriminate against me, and I received several responses from supportive individuals. This made me feel that I no longer need constant attention from my parents or those around me, as I can proactively seek information and help online.

[Participant ZX, female, 22 years]

For those with acquired vision loss (ie, vision loss that occurs after birth due to accidents, disease, or other environmental influences), the internet served as a crucial lifeline to rebuild life trajectories. As formal medical guidance on rehabilitation was often lacking, online patient communities and peer networks are usually the last resort of comfort:

Doctors usually just said, ‘there’s no treatment,’ and offered little else. It was other patients—people I met online or in hospitals—who told me about schools for the blind, massage training, or what assistive devices to get.

[Participant CT, male, 30]

Compared to traditional hospital appointment scheduling that requires in-person visits, online appointment scheduling systems have greatly improved health care access by allowing patients to register remotely via hospital WeChat Official Accounts (inside WeChat). Real-time updates offer patients more control over scheduling, allowing them to easily find alternative hospitals with available appointments.

Now, all tertiary hospitals have fully implemented online appointment systems, which is more convenient for blind people like us who could frequently use smartphones. I always make appointments through the hospital’s WeChat Official Account before seeing a doctor.

[Participant ML, female, 27 years]

#### Digital Platforms Empowering for Finding Medical Visit Companions

Hospital visits without assistance posed significant challenges for individuals with blindness, sometimes leading to delays in seeking necessary health care. For people with blindness without family or friends nearby, digital platforms offer a potential solution by connecting them with volunteer networks or organizations providing paid medical visit companions (MVCs). All participants reported benefits when receiving assistance from MVCs, as the presence of a companion alleviated anxieties and provided a sense of security throughout their hospital journey.

In the past, I would often delay medical visits because I felt overwhelmed by the hospital environment and often leave the hospital feeling that I had not addressed all my concerns, simply because I was too anxious to ask questions. When I was in Hangzhou, I began using Xianyu around one year ago to find companions. Over the past year, I have used this service a few times to arrange for someone to accompany me during medical appointments. I searched for keywords like ‘medical visit companions services’ and found options where individuals offered accompaniment services. They took me from home to the hospital and back, with charges from 30 to 80 yuan per hour. Having someone with me allows me to ask the right questions and make sure my issues are resolved.

[Participant RL, male, 26 years]

These insights highlight the empowering role that MVCs play in fostering both physical navigation and emotional support, making it easier for individuals with blindness to take charge of their health care. Through the combination of technological access and personal support, participants can be more engaged with their health care providers, which significantly improves health care seeking experience and their health outcomes.

### Exclusion: Persistent Barriers and Unmet Potentials in Digital Health Care

#### Inaccessible Online Appointment Systems

A significant challenge reported by participants was how the shift to digital platforms, while offering convenience, simultaneously erected new and formidable barriers. This dual reality was aptly summarized by a participant who noted:

Online registration/payment has made things more convenient, but there’s still a lot that’s not working.

[Participant HY, female, 24 years]

This gap was particularly evident where digital platforms, despite offering convenience, featured designs that created new exclusionary hurdles. For instance, many hospital WeChat Official Accounts, while the primary channel for online appointments, presented cluttered interfaces with complex layouts and images not optimized for screen readers. This poor usability hindered navigation and undermined informed decision-making, as 1 participant explained:

Each hospital has its own WeChat Official Account, and they differ from one another. The interface is complex, and the buttons are not designed with focus settings. This inaccessibility prevents me from accessing relevant information, thereby impacting my healthcare decision-making.

[Participant CY, female, 24 years]

#### Inaccessible Health Care Environments and Information Formats

Participants reported that complex hospital environments remain highly challenging to navigate. Standardized accessibility features—such as Braille indicators in elevators, poorly designed tactile paths, and the lack of auditory cues in key areas—are commonly not available yet. More critically, the increasing digitalization within hospitals often introduced new barriers or failed to mitigate existing physical ones.

For example, written materials such as laboratory reports, discharge records, and prescriptions are printed on paper without accessible formats like Braille or large print, making it difficult to understand and hindering patients with blindness from accessing vital information about their diagnosis and treatment. One participant expressed frustration:

Even when I get my laboratory report and discharge record, they’re just regular paper printouts with no way for me to read them independently. I feel like I’m missing out on important information, and it’s frustrating.

[Participant NX, female, 24 years]

Moreover, hospitals are increasingly relying on touchscreen-based self-service machines for tasks like registration, payment, and report retrieval, which are often inaccessible to people with blindness due to the lack of screen reader compatibility. A participant reflected on this challenge:

These machines have no screen reader compatibility, so I always need someone to briefly help me retrieve my reports.

[Participant ZY, male, 26 years]

#### Lack of Provider Competencies in Respecting Patients’ Autonomy

Many participants indicated the lack of provider competencies in respecting their autonomy, the challenge that gained particular salience within the increasingly digitized health care landscape. Specifically, a pervasive issue identified was the default assumption among many health care providers that patients with blindness lack digital literacy or the ability to independently engage with digital platforms. In an age where digital tools are designed to empower patients with greater access to information and enhanced self-management capabilities, the lack of corresponding adaptation or improvement in provider communication creates a jarring and disempowering contrast. Consequently, while most health care providers display positive attitudes, they often lack the necessary skills to effectively engage with patients who are blind. This knowledge gap can lead to communication barriers, undermining the autonomy that technology aims to support. In extreme cases, some health care staff seem to view patients with blindness as objects of curiosity rather than patients in need of medical care. A participant summed up such experiences:

Sometimes doctors ask irrelevant questions, like ‘Can you talk?’ or ‘Can you hear?’ as if they are observing an unfamiliar species instead of treating a patient. These kinds of questions only reinforce the communication barriers and make me feel like I’m not being taken seriously as a person in need of medical care, but rather as an object of curiosity.

[Participant RL, male, 26 years]

This lack of provider competencies is also reflected in the fact that health care providers often address the sighted companion instead of the patient with blindness during visits, despite the patient’s digital literacy and capacity for self-advocacy. Participants reported frequent occurrences where providers directed questions and communication to the companion, assuming they were unable to independently communicate or make decisions. One participant noted:

Whenever I have a companion, the doctor naturally chooses to speak to them instead of me. Even after repeatedly reminding the doctors that I am the patient and should be the one answering questions, they still act as if I am incapable of engaging in a normal conversation. It’s frustrating and undermines my autonomy.

[Participant ML, female, 27 years]

#### Data Privacy and Security Concerns

In the digital age, concerns regarding data privacy and information security are exacerbated for individuals with blindness, who often rely on assistive technologies and other forms of support in accessing health care. These vulnerabilities are not limited to physical interactions with medical staff but extend to broader digital infrastructures, including health platforms, mobile apps, and the public environments where these technologies are used.

Participants consistently expressed difficulties in independently navigating privacy settings or understanding consent-related information embedded within digital health applications. Complex interfaces, inaccessible terms of service, and a lack of screen reader-compatible designs hinder the ability of these individuals to make informed choices. As a participant noted:

Sometimes I just agree to everything because I can’t really read the privacy policy with the screen reader. The text layout is all over the place, and I’m not even sure what I’m consenting to.

[Participant WQ, female, 27 years]

Moreover, the use of voice-based assistive tools in public or semipublic settings presents distinct privacy risks. Given that these tools often verbalize sensitive health information, individuals in proximity may inadvertently overhear confidential data. This issue is further complicated by the involvement of MVCs, who assist with tasks such as navigating digital platforms, completing forms, or managing payments. While such assistance is often essential, it can inadvertently compromise the individuals’ sense of privacy and control. As 1 participant expressed:

Having a companion can be helpful, but sometimes I still prefer to visit alone because there are certain things I don’t want others to know. Even if I ask the volunteer to keep the information confidential and not disclose it, I still don’t feel comfortable because they have to help with payments and other tasks, and I end up feeling like I have no privacy.

[Participant CT, male, 30 years]

#### Challenges Related to the Quality and Consistency of Online Companion Support

While digital platforms offered new avenues for finding companions, this also introduced specific challenges related to the quality and consistency of support. Participants expressed concerns about the inconsistent experience and limited capabilities among MVCs, particularly regarding mobility assistance and understanding patient needs. Digital platforms often facilitated one-time interactions that lacked emotional connection and accountability, leading to varied and sometimes unreliable support:

That volunteer is in such a rush to finish his task and go home that he barely listens to what I need. There were times when I had to repeat myself multiple times just to get basic assistance.

[Participant YN, female, 27 years]

In summary, the findings highlight a complex and often contradictory landscape for educated and digitally literate young people with blindness accessing health care in the digital age. While digital platforms offer significant opportunities for empowerment in areas like appointment booking and companion support, these benefits are consistently counterbalanced by pervasive challenges such as inaccessible interfaces, systemic gaps in provider competence, and exacerbated privacy concerns. This dual reality of simultaneous empowerment and exclusion underscores the heterogeneous nature of the digital divide within vulnerable populations.

## Discussion

### Principal Findings

To the best of our knowledge, this qualitative study is the first to specifically explore the health care experiences of educated and digitally literate young people with blindness in China within the context of the rapidly evolving digital health landscape. Our findings reveal an “empowered but excluded” dynamic, a paradox that vividly illustrates the lived reality of young people with blindness as a digitally native yet vulnerable generation. On one hand, participants demonstrated that DHTs and online platforms served as valuable tools, empowering them in self-managing their health conditions, proactively accessing health care information, and efficiently finding MVCs. On the other hand, this potential for digital empowerment and enhanced independence was significantly undermined by persistent and systemic barriers. These included reduced offline access to essential services, inaccessible digital and physical health care interfaces, a pervasive lack of provider competencies in respecting patients’ autonomy within a digital context, and heightened concerns regarding data privacy and security exacerbated by digital interactions.

### Comparison With Prior Work: Empowerment

Our findings corroborate existing literature on the empowering potential of DHTs for individuals with visual impairments. Participants’ ability to effectively use online platforms for appointment booking and to access a wealth of diverse and comprehensive health information aligns with previous research highlighting improved self-management and enhanced health literacy through digital tools [36-38]. The increased autonomy and freedom participants reported, stemming from their capacity to proactively seek information and support, resonates with the broader discourse on patient empowerment in the digital age [39-42]. This study extends these insights by specifically demonstrating how educated individuals with blindness, through their active engagement with screen reader technology and other digital tools, convert these opportunities into tangible benefits, challenging simplistic narratives of universal exclusion. The use of online patient communities and peer networks to fill gaps in formal medical guidance, particularly for those with acquired vision loss, further underscores the internet’s role as a crucial lifeline and a source of social support.

A distinctive contribution of this study is the exploration of digital platforms for finding MVCs. While the importance of companions for individuals with blindness in navigating health care is well-documented [43], the use of online platforms (such as Xianyu in China) to locate and coordinate such support represents an innovative, user-driven adaptation. This strategy allows for greater independence in arranging assistance, improving the overall health care–seeking experience, an area previously underexplored in digital health literature.

### Comparison With Prior Work: Exclusion

Despite the empowering potential of DHTs, our participants’ experiences reveal a profound exclusion shaped by persistent technological disaffordances, provider interactions that often disregard patient autonomy, and digital privacy concerns, which collectively hinder their independent and equitable health care engagement. Our findings align with prior research showing that many digital health platforms remain largely inaccessible to users with blindness and low vision [44,45]. This inaccessibility manifests in specific barriers, including websites that fail to meet accessibility standards, visual-centric data displays, and complex interfaces that do not accommodate screen readers or alternative input methods [45-47]. These limitations are not just technical oversights but reflect a broader systemic neglect of the needs of people with disabilities in the design and development process.

Furthermore, our study highlights how the interaction between digital and nondigital environments can amplify existing inequalities. Beyond technological inaccessibility, participants frequently encountered health care providers who failed to recognize and respect their autonomy. This finding is consistent with previous research which shows that health care providers may hold stereotypes or paternalistic assumptions about persons who are blind, leading to exclusionary communication practices and undermining patient-centered care [43,48]. Our study adds to this discourse by illustrating how relational autonomy—a framework that emphasizes the importance of direct, respectful communication and the clinician-patient relationship as central to support patients’ identities and capabilities [49-51]. When providers fail to engage patients with blindness as active participants in their care, it not only erodes trust but also reinforces structural inequities [50,52].

Finally, our findings align with previous research showing that digital privacy poses unique challenges for users with blindness, extending beyond standard concerns about data breaches. When using visual assistance technologies or sharing sensitive data, they may be unable to independently verify what information is being disclosed [53,54]. This complexity of privacy for users with blindness is tightly interwoven with issues of accessibility, autonomy, and trust. Our study’s contribution lies in showing the compounded effect of these factors on young individuals with blindness in China, revealing that digital empowerment is fragile and easily overridden by systematic barriers within the health care environment.

### Implications for Practice and Policy

To address the systematic barriers identified in this study and improve the health care experiences of young people with blindness, we propose the following feasible policy and practical implications.

First, developers and policymakers must enforce adherence to established accessibility standards. For web-based platforms, this includes the Web Content Accessibility Guidelines [55]. However, as health care services increasingly migrate to mobile apps, it is equally critical to incorporate mobile-specific accessibility guidelines, such as Apple’s Human Interface Guidelines for accessibility [56]. Research shows that compliance is often partial; therefore, involving users with disabilities directly in a co-design process is critical for identifying specific needs, such as intuitive navigation, accessible onboarding, and the use of clear language [57].

Second, medical education and professional training must be enhanced. Evidence shows that structured communication skills training improves health care professionals’ self-efficacy and performance, leading to more effective and empathetic patient interactions. To address the biases reported by our participants, these training programs must include strategies to help providers recognize and mitigate unconscious bias related to disability, incorporating the perspectives of marginalized patient groups into the training design [58-60].

Thirdly, robust and accessible privacy controls are needed. Individuals with blindness require privacy information and controls that are both accessible and understandable, emphasizing the need for clear, multi-modal communication and cross-platform compatibility in privacy tools. The development and implementation of accessible authentication methods, such as Braille passwords or universally usable verification tools, should be prioritized.

Finally, it is crucial to empower young individuals with blindness by building their capacity for self-determination. Organizations led by and for individuals with blindness play a pivotal role in this process by equipping them with self-advocacy and daily living skills [61]. In the Chinese context, while organizations like the China Disabled Persons’ Federation provide foundational services [62], nongovernmental organizations such as the Golden Cane, the Beijing Hongdandan Cultural Service Center, and the One Plus One Disability Charity Group are vital in promoting rights advocacy and independent living skills [63-65]. A notable gap remains in dedicated health care navigation training programs that integrate digital literacy for e-health services. Closing this gap is essential to ensure young blind individuals in China can fully leverage digital health advancements.

### Strengths, Limitations, and Future Research

This study’s primary strength lies in its novel contribution to understanding the health care experiences of a previously overlooked subgroup: young, educated individuals with blindness in China. By focusing on this specific demographic, our research offers three key contributions. First, it challenges the homogeneous view of vulnerable groups by demonstrating that high-literacy individuals possess unique capabilities and face distinct challenges within the digital ecosystem. Second, it introduces and evidences the “empowered but excluded” paradox, providing a nuanced theoretical framework that moves beyond a simple narrative of digital exclusion. It shows that empowerment and exclusion are not mutually exclusive but coexist, shaped by the interplay between individual agency and systemic barriers. Third, this framework helps distinguish which challenges can be mitigated through individual effort and digital literacy versus those that require fundamental changes in policy, technology design, and clinical practice. The qualitative depth provides rich, contextualized insights that explain how and why these dynamics manifest, laying the groundwork for tailored interventions.

This study has several limitations. As a qualitative study, the findings are based on a small sample of 12 educated young individuals with blindness in China and may not be generalizable to other age groups, cultural contexts, or countries with different health care and digital infrastructures. The recruitment strategy may have introduced selection bias, potentially attracting participants with more pronounced positive or negative experiences with DHTs. Furthermore, participant recall bias might have influenced their accounts of past health care experiences. Despite these limitations, this study offers rich, contextualized insights into the lived experiences of a typically underrepresented group in digital health research. Future research should explore these issues with larger, more diverse samples, potentially using quantitative or mixed-methods approaches to assess the prevalence of the themes identified and to evaluate the effectiveness of interventions aimed at improving health care accessibility and autonomy for people with blindness in the digital age. Comparative studies across different socioeconomic and cultural settings would also be beneficial.

### Conclusions

This study explored how educated young adults with blindness in China navigate health care in the digital age, revealing an “empowered but excluded” dynamic. The potential for digital empowerment and enhanced independence, though present, is consistently curtailed by systematic barriers including inaccessible technologies, provider practices that limit patient autonomy, and privacy vulnerabilities. To bridge this gap, our findings underscore the necessity of a multifaceted approach: enhancing technological accessibility through robust standards adherence and inclusive co-design processes; improving health care provider competencies in patient-centered care via targeted training; and empowering young individuals with blindness by building their capacity for self-determination. Implementing these integrated strategies is vital for realizing equitable health care access and true independence for this digitally native yet vulnerable generation.

## Supplementary material

10.2196/79587 Multimedia Appendix 1 Interview guide.

10.2196/79587 Checklist 1 COREQ checklist.

## Abbreviations

COREQ: Consolidated Criteria for Reporting Qualitative Research
DHT: digital health technology
MVC: medical visit companion

## References

[1] Topol EJ (2019). High-performance medicine: the convergence of human and artificial intelligence. Nat Med.

[2] Chauvin J, Rispel L (2016). Digital technology, population health, and health equity. J Public Health Policy.

[3] Ahmed SK, Hussein S, Chandran D, Islam MR, Dhama K (2023). The role of digital health in revolutionizing healthcare delivery and improving health outcomes in conflict zones. Digit Health.

[4] Singh D, Nuthakki S, Naik A, Mullankandy S, Singh PK, Nuthakki Y (2022). Revolutionizing remote health: the integral role of digital health and data science in modern healthcare delivery. Cognizance J Multidiscip Stud.

[5] Mitchell M, Kan L (2019). Digital technology and the future of health systems. Health Syst Reform.

[6] Ibrahim H, Liu X, Zariffa N, Morris AD, Denniston AK (2021). Health data poverty: an assailable barrier to equitable digital health care. Lancet Digit Health.

[7] (2021). Global strategy on digital health 2020-2025. https://www.who.int/docs/default-source/documents/gs4dhdaa2a9f352b0445bafbc79ca799dce4d.pdf.

[8] van de Vijver S, Tensen P, Asiki G (2023). Digital health for all: How digital health could reduce inequality and increase universal health coverage. Digit Health.

[9] Whitehead L, Talevski J, Fatehi F, Beauchamp A (2023). Barriers to and facilitators of digital health among culturally and linguistically diverse populations: qualitative systematic review. J Med Internet Res.

[10] Yao R, Zhang W, Evans R, Cao G, Rui T, Shen L (2022). Inequities in health care services caused by the adoption of digital health technologies: scoping review. J Med Internet Res.

[11] Sieck CJ, Sheon A, Ancker JS, Castek J, Callahan B, Siefer A (2021). Digital inclusion as a social determinant of health. NPJ Digit Med.

[12] Richardson S, Lawrence K, Schoenthaler AM, Mann D (2022). A framework for digital health equity. NPJ Digit Med.

[13] Wilson S, Tolley C, Mc Ardle R (2024). Recommendations to advance digital health equity: a systematic review of qualitative studies. NPJ Digit Med.

[14] Badr J, Motulsky A, Denis JL (2024). Digital health technologies and inequalities: A scoping review of potential impacts and policy recommendations. Health Policy.

[15] Rodriguez JA, Clark CR, Bates DW (2020). Digital health equity as a necessity in the 21st century Cures Act era. JAMA.

[16] Abdul Hamid Alhassan RH, Haggerty CL, Fapohunda A, Affan NJ, Anto-Ocrah M (2025). Exploring the use of digital educational tools for sexual and reproductive health in Sub-Saharan Africa: systematic review. JMIR Public Health Surveill.

[17] Henni SH, Maurud S, Fuglerud KS, Moen A (2022). The experiences, needs and barriers of people with impairments related to usability and accessibility of digital health solutions, levels of involvement in the design process and strategies for participatory and universal design: a scoping review. BMC Public Health.

[18] Hei Y, Dolah J, Hei S (2023). A study of service touchpoints in the design of assistive products required by people with visual impairment. HumEnTech.

[19] Meade MA, Mahmoudi E, Lee SY (2015). The intersection of disability and healthcare disparities: a conceptual framework. Disabil Rehabil.

[20] Arnett JJ (2007). Emerging adulthood: What is it, and what is it good for. Child Dev Perspectives.

[21] (2022). Eye Care Indicator Menu (ECIM): A Tool for Monitoring Strategies and Actions for Eye Care Provision.

[22] Xinhua (2025). China’s internet users surpass 11 billion, powering digital economy and innovation. State Council of the People’s Republic of China.

[23] Wang S, Wang X, Zhou Y, Xu J (2022). Utilization of, satisfaction toward, and challenges for internet-based healthcare services provided by primary health institutions: Evidence from China. Front Public Health.

[24] (2025). Country map & estimates of vision loss. International Agency for the Prevention of Blindness.

[25] Sandelowski M (2000). Whatever happened to qualitative description?. Res Nurs Health.

[26] Tong A, Sainsbury P, Craig J (2007). Consolidated Criteria for Reporting Qualitative Research (COREQ): A 32-item checklist for interviews and focus groups. Int J Qual Health Care.

[27] Guest G, Bunce A, Johnson L (2006). How many interviews are enough?. Field Methods.

[28] Quadir B, Zhou M (2021). Students perceptions, system characteristics and online learning during the COVID-19 epidemic school disruption. International Journal of Distance Education Technologies.

[29] Wiranota H, Wijaya TT (2021). The international students’ perception towards online learning using the Tencent meeting during COVID-19 outbreak. J Phys Conf Ser.

[30] Buetow S (2010). Thematic analysis and its reconceptualization as “saliency analysis”. J Health Serv Res Policy.

[31] Braun V, Clarke V (2006). Using thematic analysis in psychology. Qual Res Psychol.

[32] Braun V, Clarke V (2022). Conceptual and design thinking for thematic analysis. Qual Psychol.

[33] Saunders CH, Sierpe A, von Plessen C (2023). Practical thematic analysis: a guide for multidisciplinary health services research teams engaging in qualitative analysis. BMJ.

[34] Peel K (2020). A beginners guide to applied educational research using thematic analysis. Pract Assess Res Eval.

[35] Vaismoradi M, Jones J, Turunen H, Snelgrove S (2016). Theme development in qualitative content analysis and thematic analysis. JNEP.

[36] Choi S, Nanda P, Yuen K, Ong K (2023). Bridging the gap in health literacy research: the inclusion of individuals with visual impairments. Patient Educ Couns.

[37] Taylor JJ, Subramanian A, Freitas A, Ferreira DM, Dickinson CM (2023). What do individuals with visual impairment need and want from a dialogue-based digital assistant?. Clin Exp Optom.

[38] Phochai T, Setthasuravich P, Pukdeewut A, Wetchakama S (2024). Bridging the digital disability divide: determinants of internet use among visually impaired individuals in Thailand. Disabilities.

[39] Ha S, Ho SH, Bae YH (2023). Digital health equity and tailored health care service for people with disability: user-centered design and usability study. J Med Internet Res.

[40] Hill R, Betts LR, Gardner SE (2015). Older adults’ experiences and perceptions of digital technology: (dis)empowerment, wellbeing, and inclusion. Comput Human Behav.

[41] Glicksman A (2018). Empowering older adults: opportunities and challenges. Innov Aging.

[42] Cotten SR, Zhou J, Salvendy G (2016). Human Aspects of IT for the Aged Population: Design for Aging.

[43] O’Day BL, Killeen M, Iezzoni LI (2004). Improving health care experiences of persons who are blind or have low vision: suggestions from focus groups. Am J Med Qual.

[44] Oswal SK, Oswal LM, Stephanidis C, Antona M, Ntoa S (2021). HCI International 2021—Late Breaking Posters.

[45] Lee JGW, Lee K, Lee B, Choi S, Seo J, Choe EK (2022). Personal health data tracking by blind and low-vision people: survey study. J Med Internet Res.

[46] Choi S, Chlebek CJ (2023). Exploring mHealth design opportunities for blind and visually impaired older users. Mhealth.

[47] Milne L, Bennett C, Ladner R (2014). The accessibility of mobile health sensors for blind users. J Technol Pers Disabil.

[48] Heydarian NM, Hughes AS, Morera OF, Bangert AS, Frederick AH (2021). Perspectives of interactions with healthcare providers among patients who are blind. J Blind Innov Res.

[49] Blackburn E, Durocher E, Feldman D (2019). Supporting, promoting, respecting and advocating: a scoping study of rehabilitation professionals’ responses to patient autonomy. Bioethics.

[50] Entwistle VA, Carter SM, Cribb A, McCaffery K (2010). Supporting patient autonomy: the importance of clinician-patient relationships. J Gen Intern Med.

[51] Walter JK, Ross LF (2014). Relational autonomy: moving beyond the limits of isolated individualism. Pediatrics.

[52] Martin DE, Muller E (2021). In defense of patient autonomy in kidney failure care when treatment choices are limited. Semin Nephrol.

[53] Stangl A, Sadjo E, Emami-Naeini P, Wang Y, Gurari D, Findlater L (2023). “Dump it, destroy it, send it to data heaven”: Blind people’s expectations for visual privacy in visual assistance technologies. https://dl.acm.org/doi/proceedings/10.1145/3587281.

[54] Stangl A, Shiroma K, Davis N (2022). Privacy concerns for visual assistance technologies. ACM Trans Access Comput.

[55] Li SH, Yen DC, Lu WH, Lin TL (2012). Migrating from WCAG 1.0 to WCAG 2.0—a comparative study based on web content accessibility guidelines in Taiwan. Comput Human Behav.

[56] (2025). Accessibility. Apple.

[57] Magnusson C, Hedvall PO, Caltenco H, Pissaloux E, Velazquez R (2018). Mobility of Visually Impaired People: Fundamentals and ICT Assistive Technologies.

[58] Hagiwara N, Elston Lafata J, Mezuk B, Vrana SR, Fetters MD (2019). Detecting implicit racial bias in provider communication behaviors to reduce disparities in healthcare: challenges, solutions, and future directions for provider communication training. Patient Educ Couns.

[59] Marcelin JR, Siraj DS, Victor R, Kotadia S, Maldonado YA (2019). The impact of unconscious bias in healthcare: how to recognize and mitigate it. J Infect Dis.

[60] Brand G, Bonnamy J, Dix S (2024). “You don’t see what I see”: co-designing simulation to uncover and address cognitive bias in healthcare. Med Teach.

[61] (2025). Changing what it means to be blind. Louisiana Center for the Blind.

[62] (2023). China Disabled Persons’ Federation.

[63] (2025). Disability development—committed to being a mentor. Shanghai Youren Foundation.

[64] (2025). Beijing Hongdandan Cultural Service Center.

[65] (2025). One Plus One Disability Group.

