# Supplementary material for: Health care Experiences of Educated Young Adults With Blindness in the Digital Age: Qualitative Study
Source: J Med Internet Res. 2025 Nov 21;27:e79587. doi: 10.2196/79587 (PMC12637996; doi:10.2196/79587)
Supplement: Multimedia Appendix 1 [file jmir-v27-e79587-s001.docx]

**Appendix 1: Interview guide for Educated Young Adults with Blindness**

**Part 1: Introductory Questions**

Thank you for participating in this interview. My name is [Interviewer Name], a researcher from [Institution]. This study is about understanding how you have experienced healthcare in our increasingly digital world. The interview will take about 60-90 minutes. Please be assured that your responses will be kept confidential and anonymized in our research. If at any point you feel uncomfortable or have questions, please let me know. With your permission, I would like to start recording the interview. Do I have your consent?

1.  **Personal and Educational/Professional Background:**

a.  To begin, could you please tell me a little about yourself? (Probes: Age, current occupation or field of study).

b.  What is your educational background?

2.  **Blindness and Daily Life:**

a.  Could you share some background about your blindness? (Probes: Congenital or acquired? At what age? ).

b.  How do you typically manage daily activities and independent travel? (Probes: What assistive tools or technologies, such as a guide dog, a white cane, or mobile apps, do you use?).

3.  **Digital Technology Familiarity:**

a.  How would you describe your comfort level and general use of digital technologies like smartphones, computers, and the internet in your everyday life?

b.  What specific assistive technologies or accessibility features (e.g., screen readers, voice commands, magnification software) do you regularly use to interact with digital devices?

#### **Part 2: Healthcare Experiences: The Journey Before and With Digital Tools**

1.  **Traditional Healthcare Access:**

a.  Thinking back before digital tools like patient portals or online booking were common, could you walk me through a typical experience of seeking healthcare? (Probes: How did you find a doctor, book an appointment, get to the hospital, and manage follow-up care?).

b.  What were the most significant traditional challenges you faced in those experiences as a person with blindness? (Probes: Navigating the hospital environment, communicating with staff, accessing printed medical information).

2.  **Encountering Digital Health:**

a.  When did you first start noticing digital tools (e.g., hospital websites, mobile health apps, online patient portals, telehealth consultations) becoming part of your healthcare journey?

b.  What was your initial reaction or first experience when these digital options were introduced to you?

#### **Part 3: Digital Health in Practice: Empowerment and Exclusion**

1.  **Empowerment through Digital Access (Facilitators):**

a.  In what ways, if any, have digital health tools made managing your health or accessing care easier for you? Could you provide some specific examples? (Probes: Booking appointments online, communicating with doctors via email/portals, accessing your own health records or lab results, refilling prescriptions).

b.  Can you share a story about a time when a specific digital tool or app made you feel more independent or empowered in your healthcare? What happened and how did that feel?

c.  How have your own digital skills helped you navigate or overcome accessibility issues you've encountered on these platforms?

2.  **Exclusion through Digital Barriers (Barriers):**

a.  Conversely, what are the most significant challenges or frustrations you've faced when using digital health services?

i.  (Probes: Websites or apps incompatible with your screen reader? Inaccessible online forms or documents (e.g., PDFs)? Poorly designed user interfaces? Lack of non-visual alternatives for visual information?).

b.  Can you describe a specific situation where a digital requirement created a new barrier, making you feel excluded or forcing you to rely on someone else for help?

c.  Have you ever felt that the healthcare system's push toward "digital-first" solutions assumes a level of accessibility that doesn't match your reality? Could you elaborate on that feeling?

3.  **Interaction with Healthcare Professionals in the Digital Context:**

a.  How do you perceive your interactions with doctors and nurses when digital tools are involved?

i.  (Probes: Do they seem aware of potential digital accessibility challenges for you? Do they offer accessible alternatives for information (e.g., digital text instead of printouts)? Have you ever had to educate a provider on your accessibility needs regarding digital information?).

b.  In your experience, does the use of digital health records and portals improve or complicate the communication and information sharing between you and your healthcare provider?

We’ve discussed a number of topics and ideas today. Do you have any final thoughts or things you’d like to add to our conversation?

Thank you for taking the time to share your experiences and insights.
